# Anti-Inflammatory Functions of Alverine via Targeting Src in the NF-κB Pathway

**DOI:** 10.3390/biom10040611

**Published:** 2020-04-15

**Authors:** Chae Young Lee, Han Gyung Kim, Sang Hee Park, Seok Gu Jang, Kyung Ja Park, Dong Sam Kim, Ji Hye Kim, Jae Youl Cho

**Affiliations:** 1 Department of Integrative Biotechnology, Biomedical Institute for Convergence at SKKU, Sungkyunkwan University, Suwon 16419, Korea; chaeyoung2@skku.edu (C.Y.L.); hanks523@skku.edu (H.G.K.); 2 Department of Biocosmetics, Sungkyunkwan University, Suwon 16419, Korea; 84701@naver.com; 3 Samcheok Prasiola Japonica Research Center, Samcheok City Hall, Samcheok 25914, Korea; jangsg69@korea.kr (S.G.J.); kyu5132@korea.kr (K.J.P.)

**Keywords:** anti-inflammatory effect, alverine, Src, inflammatory mediators, NF-κB

## Abstract

Alverine, a smooth muscle relaxant, is used to relieve cramps or spasms of the stomach and intestine. Although the effects of alverine on spontaneous and induced contractile activity are well known, its anti-inflammatory activity has not been fully evaluated. In this study, we investigated the anti-inflammatory effects of alverine in vitro and in vivo. The production of nitric oxide (NO) in RAW264.7 cells activated by lipopolysaccharide (LPS) or polyinosinic:polycytidylic acid (poly (I:C)) was reduced by alverine. The mRNA expression of inducible nitric oxide synthase (iNOS), cyclooxygenase-2 (COX-2), and tumor necrosis factor-α (TNF-α) was also dose-dependently inhibited by treatment with alverine. In reporter gene assays, alverine clearly decreased luciferase activity, mediated by the transcription factor nuclear factor κB (NF-κB) in TIR-domain-containing adapter-inducing interferon-β (TRIF)- or MyD88-overexpressing HEK293 cells. Additionally, phosphorylation of NF-κB subunits and upstream signaling molecules, including p65, p50, AKT, IκBα, and Src was downregulated by 200 μM of alverine in LPS-treated RAW264.7 cells. Using immunoblotting and cellular thermal shift assays (CETSAs), Src was identified as the target of alverine in its anti-inflammatory response. In addition, HCl/EtOH-stimulated gastric ulcers in mice were ameliorated by alverine at doses of 100 and 200 mg/kg. In conclusion, alverine reduced inflammatory responses by targeting Src in the NF-κB pathway, and these findings provide new insights into the development of anti-inflammatory drugs.

## 1. Introduction

Inflammation, one of the important innate immune responses, abolishes pathogens, including viruses, fungi, and bacteria, within a few minutes or hours [1,2]. However, unregulated and prolonged inflammation can evoke excessive levels of inflammatory mediators and result in resistance to apoptosis and a diminished survival advantage, even leading to carcinogenesis or multiple organ failure. For these reasons, although inflammation is a normal part of the immune system, it has to be tightly regulated [3]. Lipopolysaccharide (LPS), a Gram-negative bacterial endotoxin, is a potent activator of macrophage-derived inflammation. LPS is recognized by a complex of proteins made up of CD14, myeloid differentiation protein-2 (MD-2), and Toll-like receptor 4 (TLR4) [4]. LPS-stimulated macrophages express various inflammatory cytokines, such as tumor necrosis factor-α (TNF-α) and interleukin (IL)-1, -6, and -12, through activation of transcriptional factors nuclear factor κB (NF-κB), and activator protein-1 (AP-1) [4,5].

Src is known as one of the major proteins in the inflammatory response. Src kinase (Src) is non-receptor protein tyrosine kinase that participates in a diverse spectrum of biological responses, such as gene transcription, cell adhesion, cellular metabolism, and cell proliferation [6]. Src also plays pivotal roles in innate immunity, including recruitment and activation of immune cells, production of inflammatory cytokines, and regulation of vascular permeability [7]. For instance, phosphorylated Src activates phosphatidylinositol-3′ kinase (PI3K) by associating with the p85 subunit and AKT [8], and also increases translocation of c-Jun and p65 [9], resulting in IL-6 induction [10,11]. Therefore, Src can be a valuable target in developing promising anti-inflammatory drugs.

**Figure 1:**
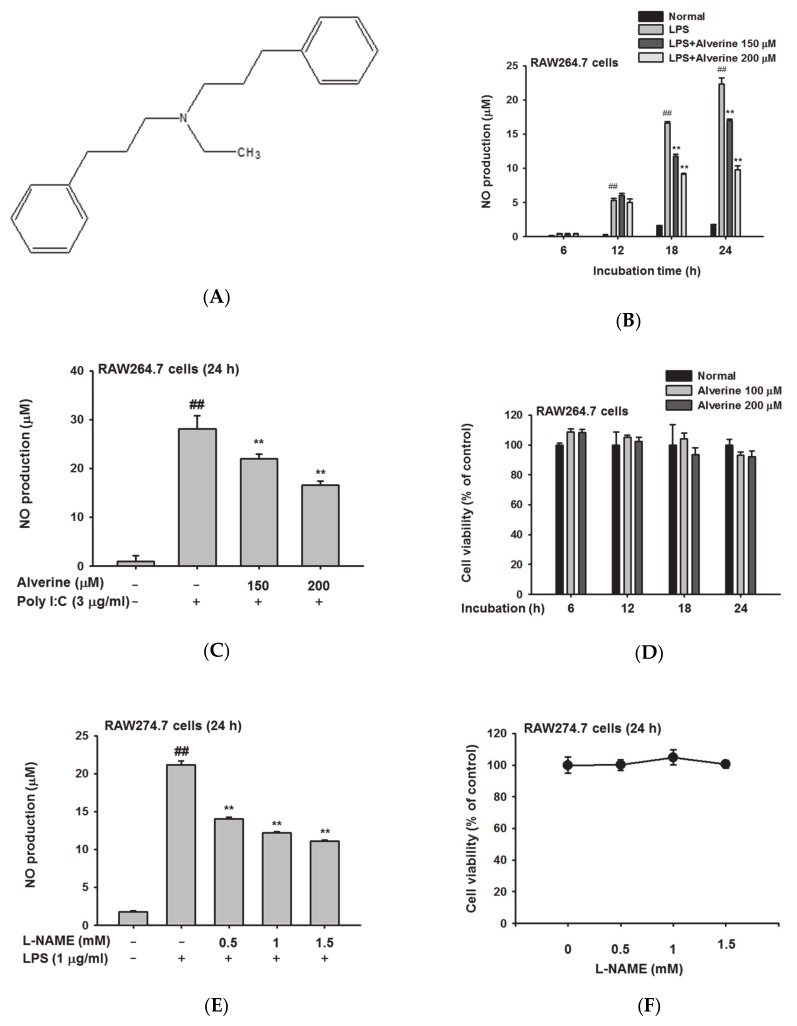
Chemical structure of alverine and its anti-inflammatory effects in activated RAW264.7 cells. (**A**) Chemical structure of alverine. (**B**,**C**) RAW264.7 cells were stimulated with lipopolysaccharide (LPS) (1 μg/mL) or polyinosinic:polycytidylic acid (poly(I:C)) (3 μg/mL) for the indicated times in the presence or absence of alverine, and NO levels were determined by Griess assay. (**D**,**F**) The viability of RAW264.7 cells treated with alverine (0–200 μM) or L-NAME (0–1.5 mM) for indicated times was measured using an MTT assay. (**E**) RAW264.7 cells were activated with LPS for 24 h in the presence of absence of L-NAME, a positive control. NO production was examined by Griess assay. All data are expressed as the mean ± standard deviation (SD) calculated from ten samples. ## *p* < 0.01 compared to the normal group, ** *p* < 0.01 compared to the control group (LPS or poly(I:C) alone).

Alverine (N-ethyl-3-phenyl-N-(3-phenylpropyl)propan-1-amine; Figure 1A) is a small molecule drug developed by Dr. Reddy’s Laboratories (UK). It is used as a smooth muscle relaxant, and acts specifically on the muscles present in locations such as the alimentary tract and uterus. [12]. Alverine regulates rectal hypersensitivity by stabilizing 5-TH1A receptors as a selective antagonist of the 5-HT1A receptor subtype [13], and proportionally regulates Ca^2+^-dependent and Ca^2+^-independent contraction in the detrusor smooth muscle [14]. Interestingly, in a previous study [15], alverine was found to be one of the components included in *Prasiola japonica* ethanol extract. According to this report, the ethanol extract of *Prasiola japonica*, also known as freshwater laver, exerts anti-inflammatory effects by reducing NO production, suggesting that alverine might be responsible. However, the pharmacological effects of alverine on inflammation are as yet poorly understood. In this study, we evaluated the potential inhibitory effects of alverine on inflammatory responses using LPS-activated macrophages in vitro and an HCl/EtOH-induced acute gastritis mouse model in vivo. Based on our findings, we propose alverine as a novel anti-inflammatory agent, enacting the anti-inflammatory principles of *Prasiola japonica*.

## 2. Materials and Methods

### 2.1. Materials

Fetal bovine serum (FBS), DMEM, and RPMI 1640 were obtained from Thermo Fisher Scientific (Waltham, MA, USA). RAW264.7 cells and HEK293 cells were purchased from ATCC (Rockville, MD, USA). MTT (3-(4,5-dimethylthiazol-2-yl)-2,5-diphenyltetrazolium bromide, a tetrazole), lipopolysaccharide (LPS), dimethyl sulfoxide (DMSO), and sodium dodecyl sulfate (SDS) were purchased from Sigma Chemical Co. (St. Louis, MO, USA). The total or phospho-specific antibodies against p50, p65, IκBα, IKKα/β, p85/PI3K, Src, Syk, AKT, HA, and β-actin were obtained from Cell Signaling Technology (Beverly, MA, USA) and Santa Cruz Biotechnology (Santa Cruz, CA, USA).

### 2.2. Cell Culture and Preparation of Drugs

RAW264.7 cells were maintained in RPMI 1640 media supplemented with 100 U/mL of penicillin/streptomycin and 10% FBS. The cells were incubated at 37 °C and 5% COHEK (human embryonic kidney). From this, 293 cells were cultured in DMEM media, supplemented with 100 U/mL of penicillin/streptomycin and 5% FBS. The cells were incubated at 37 °C and 5% CO. The stock solution (100 mM) of alverine was prepared using DMSO.

### 2.3. Determination of NO

RAW264.7 cells were plated in 96-well plates (1 × 10^6^ cells/mL) and incubated at 37 °C and 5% CO_2_ for 18 h. After incubation, the cells were treated with alverine (0 to 200 μM) for 30 min, and then further incubated with LPS (1 μg/mL) or polyinosinic:polycytidylic acid (poly(I:C)) for indicated times (6, 12, 18, and 24 h). NO production was measured using Griess reagent (0.5% naphthylethylenediamine dihydrochloride, 5% sulfanilamide, 25% H_3_PO_4_). The inhibitory effects of alverine on NO production were detected by measuring the absorbance at 540 nm using a SpectraMax 250 microplate reader.

### 2.4. Cell Viability Test

RAW264.7 cells were plated in 96-well plates (1 × 10^6^ cells/mL) and incubated at 37 °C and 5% CO_2_; for 18 h. After incubation, the cells were treated with alverine (0 to 200 μM) and incubated for indicated times (6, 12, 18, and 24 h). Next, 10 μL of MTT solution (5 mg/mL in phosphate-buffered saline, pH 7.4) was added to each well. After 3 h of incubation, 100 μL of MTT stop solution (15% SDS) was added to each well to solubilize the formazan, and the cells were incubated for 24 h, as reported previously [16]. The effects of alverine on cell viability were determined by measuring the absorbance at 570 nm using a SpectraMax 250 microplate reader.

### 2.5. mRNA Analyses Using Reverse Transcriptase Polymerase Chain Reaction

RAW264.7 cells (1 × 10^6^ cells/well) were plated on 12-well plates and treated with alverine (0 to 200 μM) for 30 min, and then further incubated with LPS (1 μg/mL) for 6 h. The medium was discarded, and the total RNA was isolated from the cells with TRIzol reagent following the manufacturer’s instructions. Using these mRNA, reverse transcription PCR was performed as previously described [17]. Briefly explained, complementary DNA (cDNA) was synthesized using a RevertAid First Strand cDNA synthesis kit (Thermo Fisher Scientific, Waltham, MA, USA). The synthesized cDNA was used for amplifying target genes with specific primers and PCRBIO HS Taq PreMix (PCR Biosystems, London, UK) in a thermal cycler (Life Technologies, Carlsbad, CA, USA). The PCR reaction was conducted with the incubation mixture (2 μL cDNA, 4 μM 5′ and 3′ primers, a 10× buffer (10 mM of Tris-HCl, pH 8.3, 50 mM of KCl, 0.1% Triton X-100), 250 μM of dNTP, 25 mM of MgCl_2_, and 1 unit of Taq) under the following incubation conditions: a 45 s denaturation time at 94 °C, an annealing time of 45 s between 55 and 60 °C, an extension time of 60 s at 72 °C, and a final extension of 7 min at 72 °C at the end of 30 cycles). The primers (Bioneer, Seoul, Korea) used in this experiment are indicated in Table 1 (F: forward, R: reverse).

### 2.6. Luciferase Reporter Gene Assay

HEK293 cells (2 × 10^5^ cells/mL) were co-transfected with either Myd88 or TRIF along with NF-κ B–Luc (luciferase) DNA and β-galactosidase, using the PEI (polyethylenimine) method in a 24-well plate. The cells were treated with alverine (0–200 μM) 24 h after transfection. After an additional 24 h, luciferase assays were performed using a Luciferase Assay System (Promega, Madison, WI, USA), as reported previously [18].

### 2.7. Preparation of Whole Cell Lysates and Western Blotting Analysis

Cells were washed with PBS once, collected, transferred to fresh tubes, and centrifuged at 12,000 rpm for 5 min at 4 °C. The cells were lysed with lysis buffer (20 mM Tris-HCl, pH 7.4; 2 mM EDTA; 2 mM ethyleneglycotetraacetic acid (EGTA); 1 mM DTT; 50 mM β-glycerol phosphate; 0.1 mM sodium vanadate; 1.6 mM pervanadate; 1% Triton X-100; 10% glycerol; 10 μg/mL aprotinin; 10 μg/mL pepstatin; 1 μM benzamide; and 2 μM PMSF). The protein lysates were then pelleted using centrifugation at 12,000 rpm for 5 min at 4 °C. The resulting supernatants were used for Western blotting analysis. Total cell lysates were prepared from RAW264.7 cells or HEK293T cells under the indicated conditions. Protein samples were separated by sodium dodecyl sulfate-polyacrylamide gel electrophoresis (Bio-Rad, Hercules, CA, USA), and immunoblotting were performed with specific antibodies, as reported previously [19]. With whole cell lysates, protein levels were examined with antibodies against the phosphorylated or total forms of AKT, IκBα, Src, p50, p65, HA, and β-actin.

### 2.8. Cellular Thermal Shift Assay

HEK293 cells (2.5 × 10^5^ cells/mL) were transfected with Src using the PEI method in a 6-well plate. DMSO or alverine (200 μM) was added to the cells 24 h after transfection, and the cells were isolated with PBS. The resuspended cells were divided equally into seven PCR tubes and heated for 3 min at 42 to 60 °C. After cooling for 3 min at 25 °C, the cells were lysed using liquid nitrogen, and this freeze–thaw cycle was repeated three times. The cell lysates were then centrifuged 13,000 rpm for 30 min at 4 °C. The protein supernatants were analyzed using Western blotting, and the intensity of the bands was quantified using ImageJ software.

### 2.9. HCl/EtOH-Induced Acute Gastritis

Five-week-old male ICR mice were purchased from ORIENT BIO (Seongnam, Korea). The mice had access to pelleted food (Samyang, Daejeon, Korea) and water ad libitum. All studies were performed according to the guidelines established by the Sungkyunkwan University Institutional Animal Care and Use Committee (Suwon, Korea; approval ID: SKKUIACUC2019-08-15-1). Inflammation of the stomach was induced with EtOH/HCl, according to a previously published method [20,21]. In brief, fasting ICR mice (*n* = 4) were treated with alverine (0–200 mg/kg) via oral route, twice per day for 2 days. Eight hours after the final injection of alverine, the mice were dosed orally with 400 μL of 70% ethanol in 150 mM HCl to induce acute gastritis. After 1 h, each mouse was anaesthetized and sacrificed using CO_2_.

### 2.10. Statistical Analysis

For the MTT and NO assays, ten wells were used in each experimental group to ensure the reliability of the results. For the luciferase assay, each experimental group has six parallel wells. In the PCR and Western blotting analysis, each experimental group was tested at least in duplicates. Gastritis in vivo experiments were performed with four mice per group. All data are expressed as the mean ± standard deviation (SD) calculated from at least four different samples. For statistical comparison, the results were analyzed using either analysis of variance (ANOVA) with the Mann–Whitney *U* test. For all analyses, *p* < 0.05 was considered statistically significant.

## 3. Results

### 3.1. Alverine Inhibited NO Production in Macrophage-Like RAW264.7 Cells

To examine the effects of alverine on macrophage-mediated inflammatory responses, NO production was first investigated in alverine-treated, RAW264.7, macrophage-like cells. NO production was induced by LPS and poly(I:C), which are TLR4 and TLR3 ligands, respectively. Alverine dose- and time-dependently blocked NO production in LPS- and poly(I:C)-stimulated RAW264.7 cells up to 61% at 200 μM under the LPS-stimulation condition, or 55% under the poly(I:C)-treated condition (Figure 1B,C). To exclude the possibility that this nitric oxide inhibitory effect was due to cytotoxicity, we evaluated the viability of RAW264.7 cells treated with alverine using an MTT assay. Alverine was not cytotoxic at any concentrations, showing less than 5% at 200 mM in 24 h-incubation conditions (Figure 1D). L-NAME, a standard anti-inflammatory compound, also showed a suppressive pattern (51% at 1.5 mM) under the same NO production conditions in LPS -treated RAW264.7 cells without cytotoxicity (Figure 1E,F).

### 3.2. Alverine Suppressed iNOS, COX-2, and TNF-α mRNA Expression through NF-κB Inhibition

**Figure 2:**
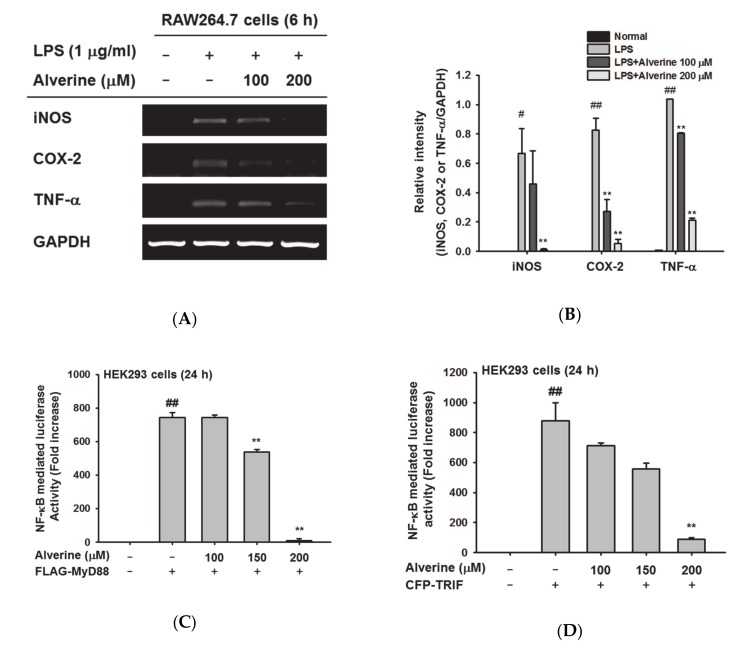

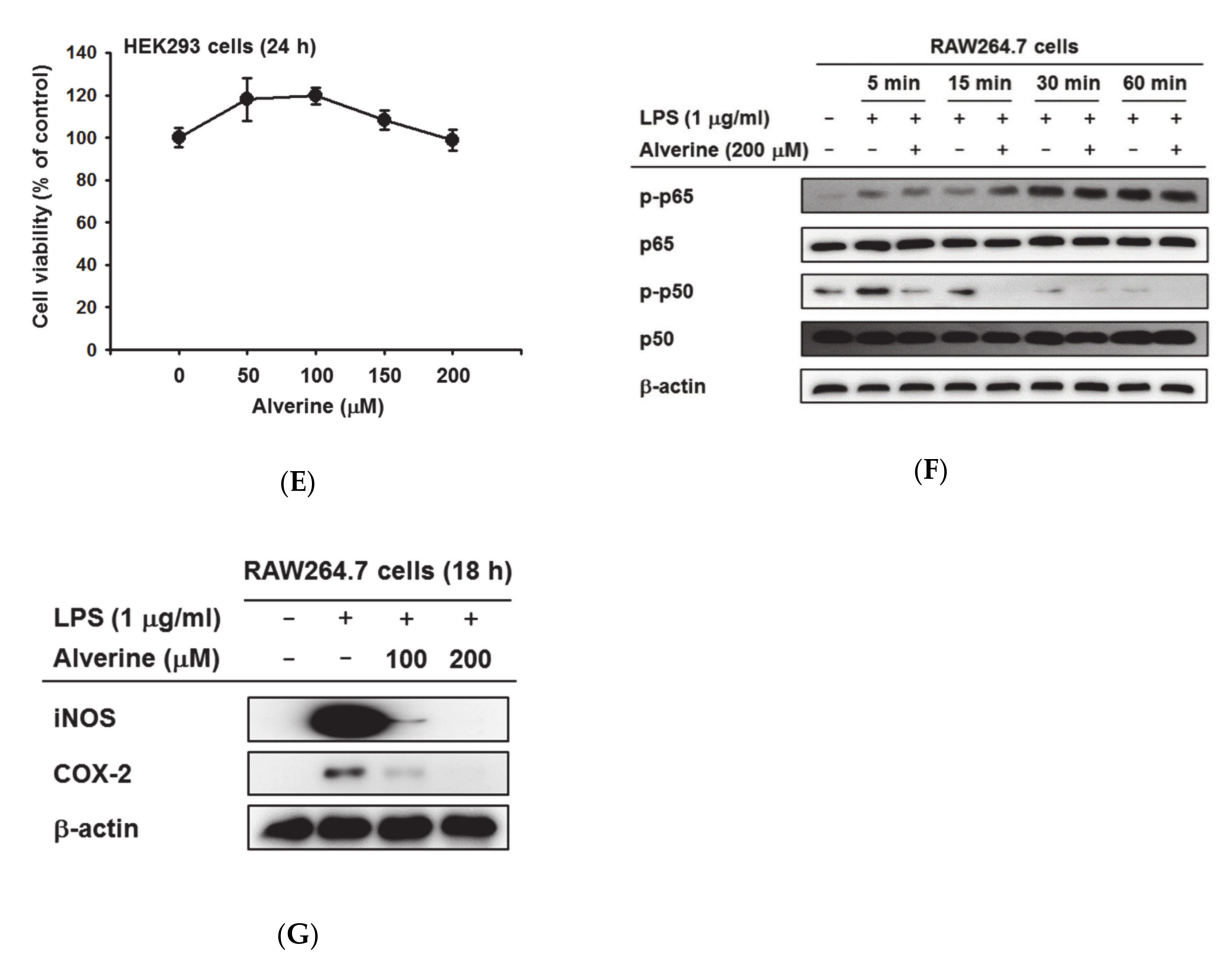
Effects of alverine on inflammatory gene expression and activation of nuclear factor κB (NF-κB). (**A**) RAW264.7 cells were treated with alverine (0–200 μM) for 30 min and then stimulated with LPS (1 μg/mL) for further 6 h. mRNA expression levels of iNOS, COX-2, and TNF-α were evaluated using reverse transcription PCR. (**B**) The relative intensity value was calculated by measuring the intensity two times, using the total levels of each protein from representative blot from a DNR bio-imaging system. GAPDH was used as control gene (**C**,**D**). NF-κB promoter binding activity was determined using a reporter gene assay in HEK293T cells. The cells were transfected with FLAG–MyD88 (**C**) or CFP–TRIF (**D**) plasmid constructs for 24 h followed by treatment with alverine (0–200 μM) for an additional 24 h. Luciferase activity was evaluated using a luminometer and normalized to that of β-galactosidase. (**E**) The viability of HEK293T cells treated with alverine (0–200 μM) was determined using the MTT assay. (**F**) The phosphorylated and total forms of NF-κB subunits (p60 and p50) were determined in LPS-stimulated RAW264.7 cells by Western blotting analysis. (**G**) RAW264.7 cells were treated with alverine (0–200 μM) and activated with LPS (1 μg/mL) for 18 h. The levels of iNOS, COX-2, and β-actin were measured using Western blotting analysis. All data (**B**–**E**) are expressed as the mean ± standard deviation (SD), calculated from two, six, or ten samples. ## *p* < 0.01 compared to the normal group, ** *p* < 0.01 compared to the control group (TRIF or MyD88 alone).

The inhibitory effects of alverine on expression levels of pro-inflammatory genes were investigated using RT-PCR analyses in LPS-stimulated RAW274.7 cells. Alverine strongly inhibited mRNA expression of iNOS, COX-2, and TNF-α in RAW274.7 cells up to 90% at 200 μM (Figure 2A,B). To understand the molecular mechanism by which alverine regulates the expression of these genes, we examined the influence of alverine on NF-κ B, an inflammatory transcriptional factor. Luciferase assays were performed to analyze the activity of NF-κB in HEK293T cells. NF-κB-mediated luciferase activity in cells transfected with the inflammation-inducing genes MyD88 or TRIF was decreased by alverine in a dose-dependent manner (Figure 2C,D). According to the MTT assay, alverine did not inhibit the viability of HEK293 cells at concentrations between 0 to 200 μM (Figure 2E), suggesting that the inhibitory effect of alverine on NF-κB–Luc is not derived from cytotoxicity. Phosphorylation levels of p50 and p65, subunits of NF-κB, were also evaluated by immunoblotting assay. Only the p-p50 level was diminished in the alverine-treated group (Figure 2F). Finally, to confirm whether inhibition of mRNA level can be also seen in the protein levels of inflammatory genes, we also check the protein level of iNOS and COX-2 by Western blotting analysis under the same condition. As Figure 2G shows, alverine clearly reduced the protein levels of these genes. Therefore, these results indicate that alverine exerts anti-inflammatory activity by specifically inhibiting NF-κB.

### 3.3. Alverine Regulated Upstream NF-κB Signaling Proteins by Targeting Src Kinase in LPS-Activated RAW264.7 Cells

**Figure 3:**
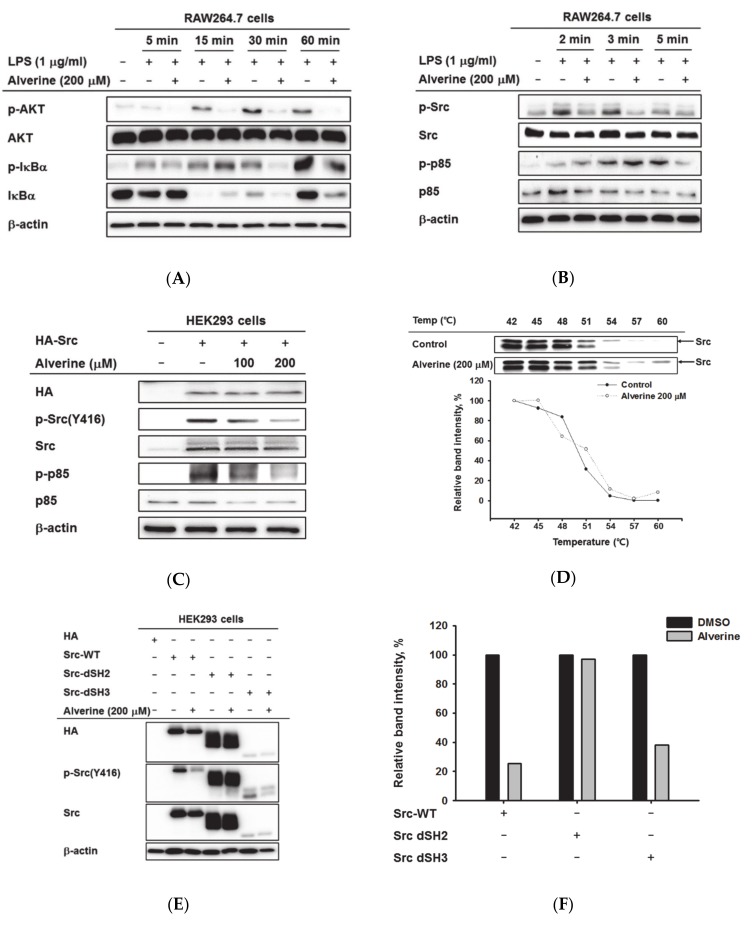
Effect of alverine on the activation of Src in NF-κB pathway. (**A**,**B**) RAW264.7 cells were treated with alverine (0–200 μM) and activated with LPS (1 μg/mL) for the indicated times. The levels of the phosphorylated and total forms of NF-κB singling proteins, including AKT, IκBα, p85, and Src, were measured using Western blotting. (**C**) HEK293T cells overexpressing HA–Src were treated with alverine (0–200 μM). The total and phosphorylated protein levels of Src, p85, HA, and β-actin were evaluated using Western blotting analysis. (**D**) A cellular thermal shift assay (CETSA) was performed with whole lysates prepared from HEK293T cells treated with alverine (200 μM) or DMSO (control). (**E**,**F**) HEK293T cells overexpressing either Src wild-type (Src-WT) or its deletion mutants without SH2 or SH3 (Src-dSH2 or Src-dSH3) were treated with alverine (200 μM). After preparing cell lysates, the total and phosphorylated protein levels of Src, HA, and β-actin were determined by Western blotting analysis. (**F**) The band intensity of p-Src in (**E**) was quantified using ImageJ. All data (**D**,**F**) are expressed as the mean calculated from duplicates.

To figure out the target molecule of the alverine, we studied the effect of alverine on the upstream signaling cascade for the activation of NF-κB in LPS-treated RAW264.7 cells. The phosphorylation of IκBα, a key regulator of NF-κB, was significantly decreased at 30 and 60 min by alverine (200 μM) (Figure 3A). Alverine also downregulated AKT phosphorylation at all time points in LPS-stimulated RAW264.7 cells (Figure 3A). It has been reported that the phosphorylation of IκBα is modulated by p85 and Src in TLR4-stimulated conditions [22,23]. Therefore, we measured the activation of p85 and Src at earlier time points. The phosphorylated p85 level in LPS-stimulated macrophages was suppressed by alverine (200 μM) at 5 min. On the other hand, alverine inhibited phosphorylation of Src kinase from 2 min after LPS stimulation (Figure 3B), suggesting that Src among the NF-κB signal proteins might be a target molecule of alverine.

To explore whether alverine can modulate Src kinase, Src was auto-phosphorylated and activated by the overexpression of the Src plasmid in HEK293T cells. As we expected, the phosphorylation of Src and p85 was clearly suppressed by alverine in a dose-dependent manner (Figure 3C). Since drug binding can lead to significant thermal stabilization of the protein, the interaction between alverine and Src was evaluated by cellular thermal shift assay (CETSA). Src kinases in the control group were mostly degraded at 57 and 60 °C, while Src was still detected in alverine-treated cells (Figure 3D). The specific domain of Src that interacts with alverine was investigated by overexpressing Src domain deletion mutants, such as those without SH2 or SH3 domains (Src-dSH2 and Src-dSH3), in HET 293T cells. Alverine-reduced Src phosphorylation induced by Src-WT and Src-dSH3, but did not affect Src phosphorylation by Src-dSH2, implying that alverine interacts with the SH2 domain of Src kinase (Figure 3E,F).

### 3.4. Alverine Exerted Anti-Inflammatory Effects in an HCL/EtOH-Induced Gastritis Mice Model

**Figure 4:**
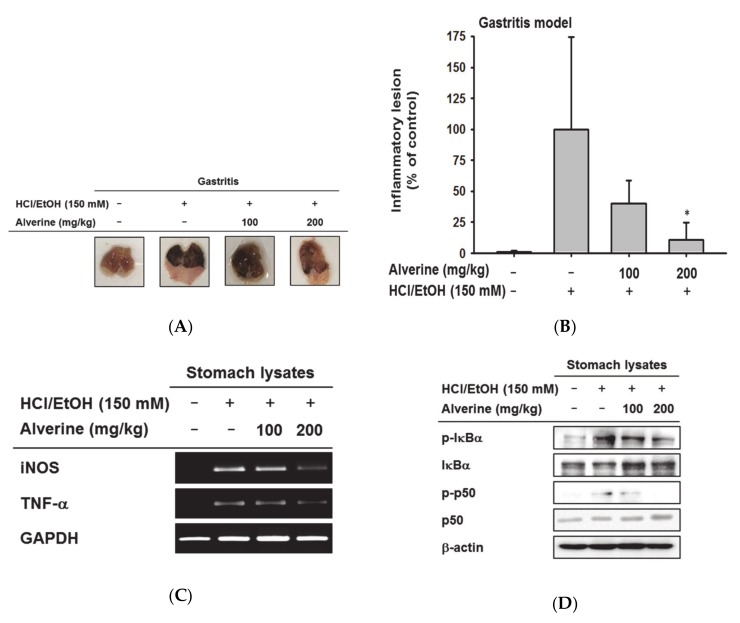
Effects of alverine on inflammatory responses in acute gastritis mouse model. (**A**,**B**) Mice were pretreated with alverine (0–200 mg/kg) orally twice per day for 3 days, and were then injected orally with EtOH/HCl (150 mM). After 1 h, mice were sacrificed, and their gastric inflammatory lesions were photographed using a digital camera. Gastritis lesions in the stomachs were quantified using ImageJ software. (**C**) mRNA expression levels of iNOS, TNF-α and GAPDH in stomach tissues were examined using RT-PCR. (**D**) Total and phosphorylated protein levels of IκBα, p50, and β-actin were analyzed using a Western blot assay. Results (B) are expressed as the mean ± standard deviation (SD), calculated from four mice. * *p* < 0.05 compared to the control group (HCl/EtOH alone).

To determine the anti-inflammatory activity of alverine in animals, in vivo gastritis was induced by injecting HCl/EtOH into mice. Alverine ameliorated the ulcerative lesions in a dose-dependent manner (Figure 4A). Similar to the in vitro results, iNOS and TNF-α mRNA levels in the stomach tissue lysates of gastritis mice were also lower in the alverine administration group (Figure 4B). Additionally, we assessed whether alverine modulated the NF-κB signaling pathway in the stomach tissues of gastritis-induced mice. Alverine clearly suppressed mRNA expression of iNOS and TNF-α under 200 mg/kg-treated conditions (Figure 4C and Supplementary Figure S1A). Finally, it was found that the levels of phosphorylated p65 and IκBα were reduced in stomach tissue lysates prepared from alverine-treated groups (Figure 4D and Supplementary Figure S1B).

## 4. Discussion

Alverine strongly inhibited the production of NO in LPS- and poly(I:C)-stimulated RAW274.7 cells without significant cytotoxicity, at concentrations up to 200 μM in dose- and time-dependent manners (Figure 1A–C). In addition, alverine decreased both mRNA expression and protein levels of inflammatory genes, such as iNOS, COX-2, and TNF-α, in LPS-stimulated RAW264.7 cells (Figure 2A). These results imply that alverine exhibits anti-inflammatory properties. In particular, alverine is expected to have a broad impact on suppressing inflammatory responses, as it reduces NO production induced by both TLR3 and TLR4.

Next, we sought to figure out the molecular mechanisms underlying the anti-inflammatory activity of alverine. It has been reported that gene expression of iNOS, COX-2, and TNF-α is mainly modulated by NF-κB and AP-1 transcriptional factors [24,25]. In addition, MyD88 and TRIF are known as essential adaptor molecules in the activation of TLR-mediated NF-κB and AP-1 signaling [26]. Therefore, NF-κB and AP-1 reporter gene assays were performed in MyD88 and TRIF-overexpressing HEK293T cells. Alverine suppressed MyD88- and TRIF-dependent NF-κB luciferase activity (Figure 2B,C, respectively), independent of the AP-1-mediated pathway (data not shown). Since phosphorylation of p65 and p50 are involved in NF-κB stabilization and promoter activity [27], p-p65 and p-p50 levels were also determined. Consistent with the reporter gene assay results, phosphorylation of p50 was also diminished by alverine. In TLR-mediated NF-κB signaling, p85-AKT pathway activation by various stimuli induces phosphorylation and degradation of IκBα, resulting in NF-κB activation [28]. Thus, we investigated the effect of alverine on LPS-induced phosphorylation of p85, AKT, and IκBα. Alverine inhibited all of these enzymes (Figure 3A,B), implying that alverine specifically regulates the NF-κB signaling pathway.

Due to the importance of NF-κB in inflammatory responses, the strategy for inhibiting this pathway has attracted attention in the development of anti-inflammatory drugs [29]. For example, glucocorticoids, such as dexamethasone and prednisone, can reduce the inflammatory response by increasing IκBα expression [30]. In addition, there are considerable reports indicating that the molecular target of nonsteroidal anti-inflammatory drugs (NSAIDs) is at least partially NF-κB [31,32,33]. In a similar vein, we concluded that alverine exerts anti-inflammatory activity through inhibition of the NF-κB pathway, and therefore may be used as an anti-inflammatory drug. Indeed, the potential of alverine as an anti-inflammatory drug was investigated in HCl/EtOH-triggered gastritis models. Alverine reduced inflammatory gene expression, as well as redness and bleeding in the gastric mucosa (Figure 4A,B). In addition, the phosphorylation of IκBα and p50 was suppressed by alverine administration in the stomach lysates of gastritis mice (Figure 4C). These results provide evidence that alverine can be used to treat inflammatory diseases by inhibiting NF-κB signaling.

Our next concern was to identify the direct targets of alverine among NF-κB signaling molecules. Treatment with LPS has long been known to cause rapid induction of tyrosine phosphorylation in macrophages [34]. It has been also reported that Src family kinases are activated after LPS treatment [35,36,37], and ligands for other TLRs besides LPS induce tyrosine phosphorylation of Src kinase substrates, such as Vav1, Pyk2, Cbl, Syk, and paxillin [38,39,40,41,42,43]. In addition, phosphorylated Src is known to induce PI3K/Akt-NF-κB pathway activation in macrophages [6]. These previous studies imply that Src kinase contributes to the activation of the LPS-induced NF-κB signaling pathway. Thus, we assessed the effect of alverine on LPS-induced Src phosphorylation. According to the immunoblotting results, alverine suppressed the phosphorylation of p85 at 5 min, but the phosphorylation of Src was inhibited starting 2 min after LPS stimulation (Figure 3B). Given the timing of responses, we suspected that Src was a target molecule of alverine. To test our hypothesis, we overexpressed Src kinase, leading to the autophosphorylation and activation of Src. As shown in Figure 3C, alverine inhibited auto-phosphorylated Src, indicating that the alverine targets Src directly, and not upstream enzymes of Src kinase. Next, CETSA analysis [44] was performed to evaluate the binding of Src and alverine in cells. CETSA is a useful method for quantifying the association of a drug with targets inside cells, on the basis of binding-induced stabilization of the protein [45]. As we expected, Src was present in a more stable form at high temperatures in the alverine treatment group compared to the control group, implying that alverine regulates Src phosphorylation and activation by binding directly to Src (Figure 3D). Src kinase is composed of four domains: the unique region and the SH3, SH2, and kinase domains [46]. To determine the alverine binding domain of Src, Src-WT, or SH2 or SH3 domain-deletion Src, they were overexpressed in HEK293T cells. The levels of phosphorylated Src in Src-dSH3 and Src-WT plasmid transfection groups were reduced by alverine; however, phosphorylation of SH2 domain-deletion Src was constant with and without alverine (Figure 3E). These data imply that alverine binds to the SH2 domain of Src and disturbs its phosphorylation, leading to regulation of inflammatory responses through modulation of the NF-κB pathway. Since NF-κB is involved in normal physiological events, such as epithelial differentiation and cell growth, long-term systemic application of a direct NF-κB inhibitor can cause significant side effects [29]. However, alverine can be expected to reduce the side effects observed in typical NF-κB inhibitors, because alverine inhibits inducible NF-κB signaling by suppressing pathogen-stimulated Src kinase rather than basal NF-κB.

## 5. Conclusions

**Figure 5:**
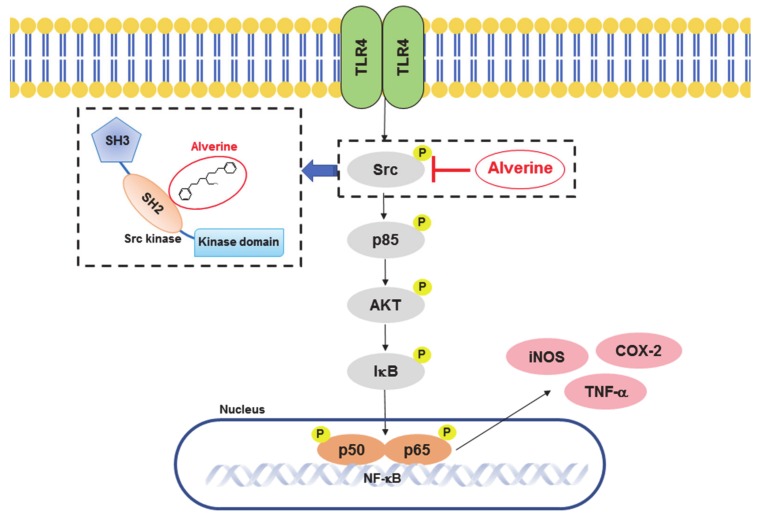
Putative inhibitory pathway for the anti-inflammatory action of alverine.

In conclusion, we have established the anti-inflammatory functions of alverine in LPS-stimulated macrophages and an in vivo gastritis model. Alverine repressed production of inflammatory mediators and the gene expression of cytokines, including iNOS, COX-2, and TNF-α, by suppressing NF-κB transcriptional activity. In addition, alverine inhibited NF-κB signaling by directly targeting Src kinase (Figure 5). Collectively, our findings suggest that alverine is a potential anti-inflammatory drug candidate with relatively fewer side effects than other NF-κB inhibitors. Although we have very clear evidence indicating that Src can be targeted by alverine, the additional possibility that this compound is able to suppress some other mechanisms involved in inflammatory responses can be considered. These could include some inhibitory actions at the translational or at the post-translational levels not yet identified in this study. Therefore, we will continue to research additional mechanisms in subsequent projects.

## Figures and Tables

**Table 1:** Sequences of PCR primers used in this study.

Targets	Direction	Sequences (5′ to 3′)
iNOS	Forward	GGAGCCTTTAGACCTCAACAGA
	Reverse	TGAACGAGGAGGGTGGTG
COX-2	Forward	CACTACATCCTGACCCACTT
	Reverse	ATGCTCCTGCTTGAGTATGT
TNF-α	Forward	GCCTCTTCTCATTCCTGCTTG
	Reverse	CTGATGAGAGGGAGGCCATT
GAPDH	Forward	CAATGAATACGGCTACAGCAAC
	Reverse	AGGGAGATGCTCAGTGTTGG

## Supplementary Materials

The following are available online at https://www.mdpi.com/2218-273X/10/4/611/s1, Figure S1: Anti-inflammatory effects of alverine in an in vivo acute gastritis model.

## Abbreviations

PAMPs	Pathogen-associated molecular patterns
PRRs	Pattern recognition receptors
TLRs	Toll-like receptors
MYD88	Myeloid differentiation primary response 88
TRIF	TIR-domain-containing adapter-inducing interferon-β
COX-2	Cyclooxygenase-2
IL	Interleukin
NO	Nitric oxide
Pred	Prednisolone
TNF-α	Tumor necrosis factor-alpha
AP-1	Activator protein 1
IP	Intraperitoneal
NOS	NO synthases

## References

[1] Newton K., Dixit V.M. (2012). Signaling in innate immunity and inflammation. Cold Spring Harb. Perspect. Biol..

[2] Kim J.H., Yi Y.S., Kim M.Y., Cho J.Y. (2017). Role of ginsenosides, the main active components of Panax ginseng, in inflammatory responses and diseases. J. Ginseng Res..

[3] Shacter E., Weitzman S.A. (2002). Chronic inflammation and cancer. Oncology.

[4] Fujihara M., Muroi M., Tanamoto K., Suzuki T., Azuma H., Ikeda H. (2003). Molecular mechanisms of macrophage activation and deactivation by lipopolysaccharide: Roles of the receptor complex. Pharmacol. Ther..

[5] Guha M., Mackman N. (2001). LPS induction of gene expression in human monocytes. Cell Signal..

[6] Byeon S.E., Yi Y.-S., Oh J., Yoo B.C., Hong S., Cho J.Y. (2012). The role of Src kinase in macrophage-mediated inflammatory responses. Mediators Inflamm..

[7] Okutani D., Lodyga M., Han B., Liu M. (2006). Src protein tyrosine kinase family and acute inflammatory responses. Am. J. Physiol. Lung Cell Mol. Physiol..

[8] Pleiman C.M., Hertz W.M., Cambier J.C. (1994). Activation of phosphatidylinositol-3′kinase by Src-family kinase SH3 binding to the p85 subunit. Science.

[9] Kim E., Yi Y.S., Son Y.J., Han S.Y., Kim D.H., Nam G., Hossain M.A., Kim J.H., Park J., Cho J.Y. (2018). BIOGF1K, a compound K-rich fraction of ginseng, plays an antiinflammatory role by targeting an activator protein-1 signaling pathway in RAW264.7 macrophage-like cells. J. Ginseng Res..

[10] Tang C.-H., Hsu C.-J., Yang W.-H., Fong Y.-C. (2010). Lipoteichoic acid enhances IL-6 production in human synovial fibroblasts via TLR2 receptor, PKCδ and c-Src dependent pathways. Biochem. Pharmacol..

[11] Lee J.O., Choi E., Shin K.K., Hong Y.H., Kim H.G., Jeong D., Hossain M.A., Kim H.S., Yi Y.S., Kim D. (2019). Compound K, a ginsenoside metabolite, plays an antiinflammatory role in macrophages by targeting the AKT1-mediated signaling pathway. J. Ginseng Res..

[12] Mitchell S., Mee A., Smith G., Palmer K., Chapman R. (2002). Alverine citrate fails to relieve the symptoms of irritable bowel syndrome: Results of a double-blind, randomized, placebo-controlled trial. Aliment. Pharmacol. Ther..

[13] Coelho A.M., Jacob L., Fioramonti J., Bueno L. (2001). Rectal antinociceptive properties of alverine citrate are linked to antagonism at the 5-HT1A receptor subtype. J. Pharm. Pharmacol..

[14] Hayase M., Hashitani H., Suzuki H., Kohri K., Brading A. (2007). Evolving mechanisms of action of alverine citrate on phasic smooth muscles. Br. J. Pharmacol..

[15] Seo D.W., Kim H.J., Jang S.K., Jun M., Joo S.S. (2013). Screening of functional components derived from fresh water laver, Prasiola japonica, and its pharmacological properties. J. Biomed. Res..

[16] Han S.Y., Kim J., Kim E., Kim S.H., Seo D.B., Kim J.H., Shin S.S., Cho J.Y. (2018). AKT-targeted anti-inflammatory activity of Panax ginseng calyx ethanolic extract. J. Ginseng Res..

[17] Baek K.-S., Yi Y.-S., Son Y.-J., Yoo S., Sung N.Y., Kim Y., Hong S., Aravinthan A., Kim J.-H., Cho J.Y. (2016). In vitro and in vivo anti-inflammatory activities of Korean Red Ginseng-derived components. J. Ginseng Res..

[18] Jung K.K., Lee H.S., Cho J.Y., Shin W.C., Rhee M.H., Kim T.G., Kang J.H., Kim S.H., Hong S., Kang S.Y. (2006). Inhibitory effect of curcumin on nitric oxide production from lipopolysaccharide-activated primary microglia. Life Sci..

[19] Burnette W.N. (1981). “Western blotting”: Electrophoretic transfer of proteins from sodium dodecyl sulfate-polyacrylamide gels to unmodified nitrocellulose and radiographic detection with antibody and radioiodinated protein A. Anal. Biochem..

[20] Yang Y., Yang W.S., Yu T., Sung G.-H., Park K.W., Yoon K., Son Y.-J., Hwang H., Kwak Y.-S., Lee C.-M. (2014). ATF-2/CREB/IRF-3-targeted anti-inflammatory activity of Korean red ginseng water extract. J Ethnopharmacol..

[21] Lee S.-J., Park J.-Y., Choi K.-S., Ock C.-Y., Hong K.-S., Kim Y.-J., Chung J.-W., Hahm K.-B.J. (2010). Efficacy of Korean red ginseng supplementation on eradication rate and gastric volatile sulfur compound levels after Helicobacter pylori eradication therapy. J. Ginseng Res..

[22] Li X., Tupper J.C., Bannerman D.D., Winn R.K., Rhodes C.J., Harlan J.M. (2003). Phosphoinositide 3 kinase mediates Toll-like receptor 4-induced activation of NF-kappa B in endothelial cells. Infect. Immun..

[23] Lee H.S., Moon C., Lee H.W., Park E.M., Cho M.S., Kang J.L. (2007). Src tyrosine kinases mediate activations of NF-kappaB and integrin signal during lipopolysaccharide-induced acute lung injury. J. Immunol..

[24] Makarov S.S. (2000). NF-kappaB as a therapeutic target in chronic inflammation: Recent advances. Mol. Med. Today.

[25] Schonthaler H.B., Guinea-Viniegra J., Wagner E.F. (2011). Targeting inflammation by modulating the Jun/AP-1 pathway. Ann. Rheum. Dis..

[26] Takeda K., Akira S. (2004). TLR signaling pathways. Semin. Immunol..

[27] Christian F., Smith E.L., Carmody R.J. (2016). The Regulation of NF-kappaB Subunits by Phosphorylation. Cells.

[28] Bai D., Ueno L., Vogt P.K. (2009). Akt-mediated regulation of NFkappaB and the essentialness of NFkappaB for the oncogenicity of PI3K and Akt. Int. J. Cancer.

[29] Yamamoto Y., Gaynor R.B. (2001). Therapeutic potential of inhibition of the NF-kappaB pathway in the treatment of inflammation and cancer. J. Clin. Invest..

[30] Auphan N., DiDonato J.A., Rosette C., Helmberg A., Karin M. (1995). Immunosuppression by glucocorticoids: Inhibition of NF-kappa B activity through induction of I kappa B synthesis. Science.

[31] Pierce J.W., Read M.A., Ding H., Luscinskas F.W., Collins T. (1996). Salicylates inhibit I kappa B-alpha phosphorylation, endothelial-leukocyte adhesion molecule expression, and neutrophil transmigration. J. Immunol..

[32] Yin M.J., Yamamoto Y., Gaynor R.B. (1998). The anti-inflammatory agents aspirin and salicylate inhibit the activity of I(kappa)B kinase-beta. Nature.

[33] Yamamoto Y., Yin M.J., Lin K.M., Gaynor R.B. (1999). Sulindac inhibits activation of the NF-kappaB pathway. J. Biol. Chem..

[34] Weinstein S.L., Gold M.R., DeFranco A.L. (1991). Bacterial lipopolysaccharide stimulates protein tyrosine phosphorylation in macrophages. Proc. Natl. Acad. Sci. USA.

[35] Boulet I., Ralph S., Stanley E., Lock P., Dunn A.R., Green S.P., Phillips W.A. (1992). Lipopolysaccharide- and interferon-gamma-induced expression of hck and lyn tyrosine kinases in murine bone marrow-derived macrophages. Oncogene.

[36] Beaty C.D., Franklin T.L., Uehara Y., Wilson C.B. (1994). Lipopolysaccharide-induced cytokine production in human monocytes: Role of tyrosine phosphorylation in transmembrane signal transduction. Eur. J. Immunol..

[37] Stefanova I., Corcoran M.L., Horak E.M., Wahl L.M., Bolen J.B., Horak I.D. (1993). Lipopolysaccharide induces activation of CD14-associated protein tyrosine kinase p53/56lyn. J. Biol. Chem..

[38] Hazeki K., Masuda N., Funami K., Sukenobu N., Matsumoto M., Akira S., Takeda K., Seya T., Hazeki O. (2003). Toll-like receptor-mediated tyrosine phosphorylation of paxillin via MyD88-dependent and -independent pathways. Eur. J. Immunol..

[39] Stovall S.H., Yi A.K., Meals E.A., Talati A.J., Godambe S.A., English B.K. (2004). Role of vav1- and src-related tyrosine kinases in macrophage activation by CpG DNA. J. Biol. Chem..

[40] Achuthan A., Elsegood C., Masendycz P., Hamilton J.A., Scholz G.M. (2006). CpG DNA enhances macrophage cell spreading by promoting the Src-family kinase-mediated phosphorylation of paxillin. Cell. Signal..

[41] Orlicek S.L., Hanke J.H., English B.K. (1999). The src family-selective tyrosine kinase inhibitor PP1 blocks LPS and IFN-gamma-mediated TNF and iNOS production in murine macrophages. Shock.

[42] Scholz G., Cartledge K., Dunn A.R. (2000). Hck enhances the adherence of lipopolysaccharide-stimulated macrophages via Cbl and phosphatidylinositol 3-kinase. J. Biol. Chem..

[43] Williams L.M., Ridley A.J. (2000). Lipopolysaccharide induces actin reorganization and tyrosine phosphorylation of Pyk2 and paxillin in monocytes and macrophages. J. Immunol..

[44] Molina D.M., Jafari R., Ignatushchenko M., Seki T., Larsson E.A., Dan C., Sreekumar L., Cao Y., Nordlund P. (2013). Monitoring drug target engagement in cells and tissues using the cellular thermal shift assay. Science.

[45] Boggon T.J., Eck M.J. (2004). Structure and regulation of Src family kinases. Oncogene.

[46] Gilmore T.D., Herscovitch M. (2006). Inhibitors of NF-kappaB signaling: 785 and counting. Oncogene.

