# Supplementary material for: Anti-Inflammatory Functions of Alverine via Targeting Src in the NF-κB Pathway
Source: Biomolecules. 2020 Apr 15;10(4):611. doi: 10.3390/biom10040611 (PMC7225962; doi:10.3390/biom10040611)
Supplement: Supplementary file 1 [file biomolecules-10-00611-s001.pdf]

Supplementary materials

Anti-inflammatory functions of alverine via targeting Src in the NF-κB pathway

Chae Young Lee [1], Han Gyung Kim [1], Sang Hee Park [2], Seok Gu Jang [3], Kyung Ja Park [3], Dong Sam Kim [3,*], Ji Hye Kim [1,*] and Jae Youl Cho [1,2,*]

1 Department of Integrative Biotechnology, Biomedical Institute for Convergence at SKKU, Sungkyunkwan University, Suwon 16419, Republic of Korea; chaeyoung2@skku.edu (C.Y.L.), hanks523@skku.edu (H.G.K.), kjhmlkjhml@hanmail.net (J.H.K.), and jaecho@skku.edu (J.Y.C.)

2 Department of Biocosmetics, Sungkyunkwan University, Suwon 16419, Republic of Korea; 84701@naver.com (S.H.P.)

3 Samcheok Prasiola Japonica Research Center, Samcheok City Hall, Samcheok 25914, Republic of Korea; prasiolra@korea.kr (D.S.K.); jangsg69@korea.kr (S.J.); kyu5132@korea.kr (K.J.P.)

* Correspondence: jaecho@skku.edu (J.Y.C.); +82-31-290-7868 (J.Y.C.), kjhmlkjhml@hanmail.net (J.H.K.); +82-82-31-290-7878 (J.H.K.), and prasiolra@korea.kr (D.S.K.); (+82) 33-570-3337 (D.S.K.)

Supplementary Figure S1. Anti-inflammatory effects of alverine in an *in vivo* acute gastritis model. Mice were pretreated with alverine (0–200 mg/kg) orally twice per day for 3 days then injected orally with EtOH/HCl (150 mM). (A) mRNA expression levels of iNOS, TNF-α and GAPDH in stomach tissues were examined using RT-PCR. (B) Total and phosphorylated protein levels of IκBα, p65, p50, iNOS, and β-actin were analyzed using a Western blot analysis.

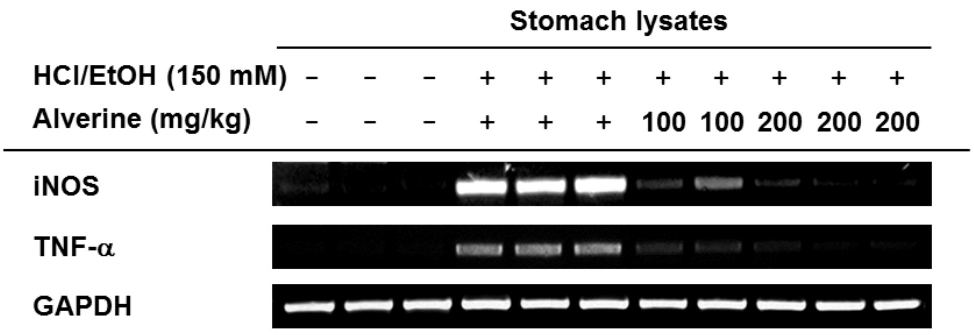

(A)

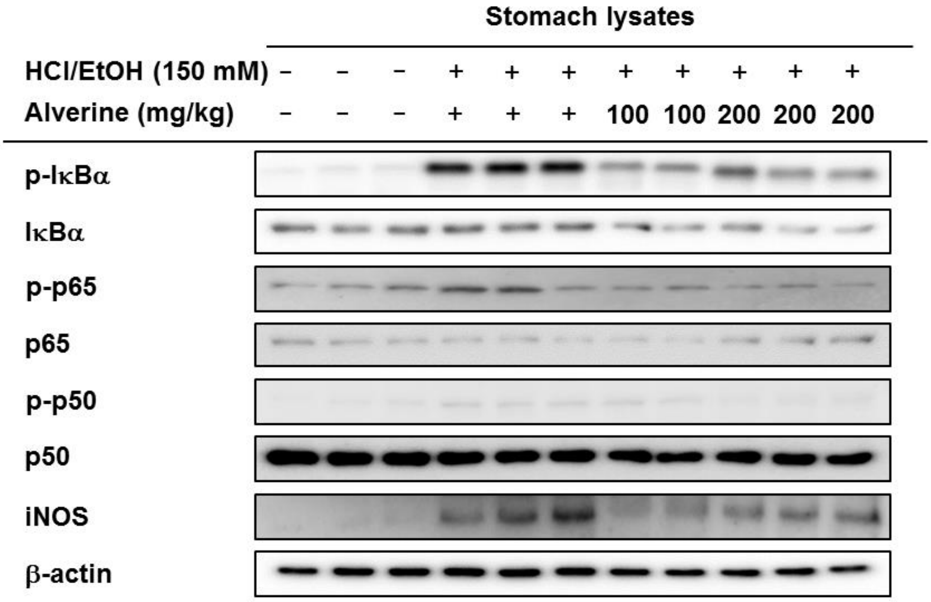

Stomach lysates
HCl/EtOH (150 mM) – – – + + + + + + + +
Alverine (mg/kg) – – – + + + 100 100 200 200 200
p-IκBα
IκBα
p-p65
p65
p-p50
p50
iNOS
β-actin

(B)
